# Preliminary Investigation of Poly-Ether-Ether-Ketone Based on Fused Deposition Modeling for Medical Applications

**DOI:** 10.3390/ma11020288

**Published:** 2018-02-12

**Authors:** Feng Zhao, Dichen Li, Zhongmin Jin

**Affiliations:** 1 State Key Laboratory for Manufacturing System Engineering, School of Mechanical Engineering, Xi’an Jiaotong University, Xi’an 710054, China; summercoming@stu.xjtu.edu.cn (F.Z.); zmjin@mail.xjtu.edu.cn (Z.J.); 2 Institute of Medical and Biological Engineering, University of Leeds, Leeds LS2 9JT, UK

**Keywords:** FDM, PEEK, mechanical strength, FTIR, cytotoxicity test, medical application

## Abstract

Poly-ether-ether-ketone (PEEK) fabricated by fused deposition modeling for medical applications was evaluated in terms of mechanical strength and in vitro cytotoxicity in this study. Orthogonal experiments were firstly designed to investigate the significant factors on tensile strength. Nozzle temperature, platform temperature, and the filament diameter were tightly controlled for improved mechanical strength performance. These sensitive parameters affected the interlayer bonding and solid condition in the samples. Fourier transform infrared (FTIR) spectrometry analysis was secondly conducted to compare the functional groups in PEEK granules, filaments, and printed parts. In vitro cytotoxicity test was carried out at last, and no toxic substances were introduced during the printing process.

## 1. Introduction

As an important member of polyaryletherketone (PAEK) family, poly-ether-ether-ketone (PEEK) is an exceptional material with many excellent characteristics that are potentially suitable for medical applications. A stable aromatic structure makes PEEK available for repeatable sterilization [1], which is ideal for surgical instruments and dental devices. Furthermore, in vivo biocompatibility and stability demonstrate PEEK to be an implantable material, and an increased number of applications can be expected. Excellent mechanical properties, an elastic modulus similar to cortical bone, and a unique ability to be analyzed and imaged by a variety of techniques, including X-rays, magnetic resonance imaging (MRI), and computer tomography (CT), together with elimination of metallic ions releasing, attribute PEEK as an alternative material to metals in the orthopedic field [2,3].

It is an increasing trend that orthopedic implants are customized to adapt to individual differences. Additive manufacturing (AM), a layer-by-layer fabricating method, because of its low cost, short production time, and feasibility of complex architecture, is popular for fabricating patient-specific implants [4]. Currently, there are limited standards for PEEK fabricated via AM; however, criteria for PEEK fabricated via machining, extruding, and compression molding are often referred to. A series of physical (such as tensile and flexural properties), chemical (such as infrared spectrum), and biological tests have been recommended when virgin PEEK is used for medical devices [5,6].

For high melting temperature materials, such as PEEK, selective laser sintering (SLS) and fused deposition modeling (FDM) are two available fabrication methods. More economical than SLS, FDM has increasingly been attempted for PEEK manufacturing in recent years.

The feasibility of PEEK printed via FDM was firstly verified by Valentan [7] in 2013. A special FDM machine with a controlled nozzle temperature and a constant ambient temperature (up to 300 °C) was developed for materials extrusion. Moreover, tensile tests were performed for evaluating the processing method. Wu et al. [8,9] also designed a custom-made FDM printer for PEEK printing. The temperatures of nozzle, platform, and chamber were all controlled. The effect of the nozzle and chamber temperature on the deformation was firstly investigated. Mechanical strength was further optimized in terms of layer thickness as well as raster orientation. A filament-based extrusion system was a better option rather than a syringe-based system, as reported by Vaezi [10]. In that study, the nozzle temperature could reach up to 460 °C, and material degradation at different nozzle temperatures and the fracture mode of the specimens printed by different raster angles were discussed. Platform and ambient temperatures were set constantly at 130 °C and 80 °C respectively during the printing. Yang et al. [11] investigated the relationship between crystallinity and mechanical properties. They pointed out that proper thermal controls, both the printing temperature and heat post-treatment, were helpful in achieving different crystallinities for different parts, or in different regions in the same part. Thus, various mechanical properties were achieved. According to previous literature, the nozzle, platform, and ambient temperatures are three important thermal parameters during printing. However, these previous investigations concerning the effects of thermal parameters have mainly focused on part deformation or materials degradation, while research concerning the optimization of mechanical properties have been mainly in terms of non-thermal parameters, such as layer thickness and raster angle. The relationship between thermal factors and mechanical strength remains unclear.

The objective of this research was to verify that PEEK processed via FDM can feasibly be applied to medical fields, not only in terms of mechanical strength but also in terms of toxic substances introduced during extrusion. In this study, all temperature-related parameters during FDM printing—nozzle temperature, ambient temperature, and platform temperature—were chosen as input factors for mechanical strength optimization. Moreover, samples printed at the upper limit of the temperatures were tested and examined for degradation and potential toxic substances.

## 2. Materials and Method

### 2.1. Filament Preparation and Printer Design

**Figure 1:**
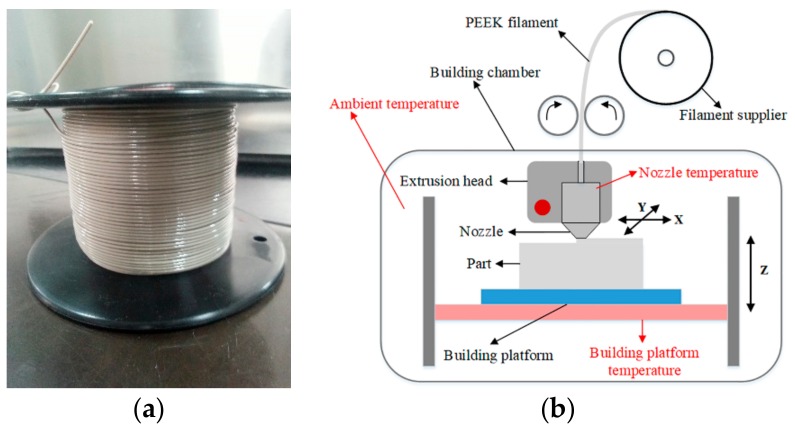
The filament and fused deposition modeling (FDM) printer: (**a**) Poly-ether-ether-ketone (PEEK) filament extruded in the lab. (**b**) The homemade FDM printer for PEEK: the temperature of the nozzle, the building platform, and the ambient can be controlled.

The filament was produced from PEEK granules (VICTREX^®^, 450G, Thornton Cleveleys, UK) by a standalone extruder in our laboratory. To avoid bubbles in the filament, PEEK granules were dried for more than 5 h at 120 °C in an oven to ensure a residual moisture below 0.1%. The dried granules were then fed into the extruder. The diameter of the filament was controlled by the difference between extrusion and take-off wind-up speeds; a faster extrusion speed led to a diameter increase, and a faster take-off wind-up speed led to a diameter decrease. The nominal diameter was set at 1.75 mm. An air cooling device, instead of a water bath, was adopted to prevent bubbles from forming and to avoid a large diameter change. It was necessary to keep the moisture of the filament under 0.1% during the printing procedure. The final filament used is shown in Figure 1a.

A homemade FDM printer, as shown in Figure 1b, was used for PEEK printing. A heated building plate was adopted to improve the binding between the platform and the initial layer. Furthermore, a sealed building chamber with heaters was beneficial for warping improvement. The peak temperatures of the nozzle, the building plate, and the ambient were 375 °C, 270 °C, and 170 °C, respectively.

### 2.2. Significance Analysis of Thermo-Parameters

The effect of the thermal parameters on the mechanical strength was evaluated by a L25 (5^3^) orthogonal experiment. The temperatures of the nozzle ($T_{N}$), the building plate ($T_{P}$), and the building chamber ($T_{E}$) were three thermo-parameters to be controlled during the printing. Thus, these variables were chosen as impact factors with five levels, from 355 to 375 °C with a 5 °C increment, from 230 to 270 °C with a 10 °C increment, and from 130 to 170 °C with a 10 °C increment, respectively. Levels of the impact factors were chosen based on preliminary trials in the laboratory. Tensile strength was employed as an evaluation index for the orthogonal experiments.

**Figure 2:**
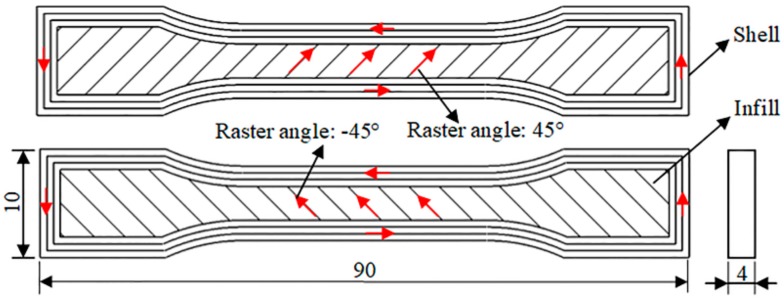
Samples for tensile tests and details of the processing path.

Dog bone bar specimens in 1BA type specified by ISO 527, as shown in Figure 2, were employed for the tensile test, printed with a 0.40 mm nozzle size, a 0.20 mm layer thickness, a 0.60 mm out shell thickness, a 100% infill density, a 30 mm/s printing speed, and an alternating +45°/−45°raster angle. Details of experiment groups setting are listed in Appendix A. Tensile strength tests were carried out at a 1 mm/s loading speed at room temperature (25 °C) by a mechanical testing machine (CMT4304, MTS Corp, Eden Prairie, MN, USA). Five specimens for each group were tested for statistical consideration.

Based on orthogonal experiments, the analysis of variance (ANOVA) was performed to reveal the significance of thermal parameters with respect to tensile strength. In ANOVA, the sum of squares (SS) represented a measure of variation from the mean, some of which came from the thermo-factors and the remaining from random factors or error. Mean squares (MS) represented an estimate of population variance, which was calculated by dividing the corresponding SS by the degrees of freedom. F value was a direct parameter to determine whether a factor was significant by comparing this ratio against a critical value in the F-distribution table, which was calculated by dividing the MS of every factor by the MS of the error [12].

### 2.3. FTIR

As the heating process existed in the extrusion stage from PEEK granules into filament and the printing process, the bonds of the atoms in the material might have changed. Thus, the original PEEK granules, the PEEK filament, and the PEEK samples printed at the highest nozzle temperature 375 °C were used for Fourier transform infrared (FTIR) spectrometry analyses to quantify material degradation.

A Nicolet iS10 FTIR spectrometer assembled with an attenuated total reflectance (ATR) cell was used to measure the spectra. The filament was broken into small pellets of around 2 mm, like the original PEEK granules. One layer of the printed part was stripped and used for the FTIR test. All spectra were collected from the 550 to 4000 cm^−1^ bands of over 600 scans with a resolution of 1 cm^−1^. OMNIC software (OMNIC 9.7.7, Thermo Fisher Scientific, Waltham, MA, USA) was employed to record the spectra, correct the variation, and reduce the noise. Each specimen was tested three times.

### 2.4. Test for In Vitro Cytotoxicity

As the in vitro cytotoxicity test is the most basic evaluation when PEEK is used as medical materials according to the ISO standards. In this part, PEEK samples related to FDM processing were evaluated, mainly based on ISO 10993: Biological Evaluation of Medical Devices—Part 5: Tests for In Vitro Cytotoxicity.

Three blocks (25 mm × 15 mm × 10 mm) were printed at the ceiling set-up of the printer ($T_{N\;}$ = 375 °C, $T_{P}\;$ = 270 °C, and $T_{E\;}$ = 170 °C). Samples were firstly soaked in ethanol and 75% alcohol for 8 h and 2 h, respectively, dried, and finally sterilized by ultraviolet radiation for 8 h.

After the treatment, samples were incubated in DMEM (Dulbecco’s modified Eagle’s medium) with 5% fetal bovine serum for 24 h and with a mass/volume ratio of 0.1 g/mL in a humidified atmosphere of 5% CO2, 95% air at 37 °C. Partial extract was then diluted to 75%, 50%, and 25% by adding culture medium (DMEM with 5% fetal bovine).

High density polyethylene extract, culture medium with 0.64% phenol, and fresh culture medium with 5% fetal bovine serum were used for the negative control group, the positive control group, and the blank control group, respectively.

The L929 fibroblast cell line in the logarithmic growth phase was employed for the cytotoxicity assay. The cell line was obtained from the State Key Laboratory for Manufacturing System Engineering (Xi’an, China). L929 cell suspension with a 10^5^ cells/mL concentration was firstly seeded into 96-well plates (100 μL/well) and incubated for 24 h. Secondly, the cultural medium was substituted with 100 μL of extracts with different concentrations, the negative control medium, the positive control medium, and the blank control medium, respectively, and put into an incubator for 24 h. After the incubation, cell morphology was examined under a microscope (A1, Nikon, Tokyo, Japan). In the end, the medium was replaced with 100 μL of fresh medium and 10 μL of CCK8 solution and incubated for 3 h. The cell viability was calculated based on the absorbance at 450 nm by a microplate reader (MULTISKAN GO, Thermo Fisher Scientific, Waltham, MA, USA) using the following equation:

$$\mathrm{Cell}\;\mathrm{viability}\;=\;\frac{A_{s}{\;-\;A}_{b}}{A_{c}{\;-\;A}_{b}}\;\times \;100\%$$

where $A_{s}$ is the absorbance of the test groups, which includes cells, the culture medium, CCK-8, and the extracts with different concentrations; $A_{b}$ is the absorbance of the control group, which includes cells, the culture medium, and CCK-8; $A_{c}$ is the absorbance of the blank group, which includes the culture medium and CCK-8.

## 3. Results and Discussion

### 3.1. Significance Analysis of Thermo-Parameters

**Figure 3:**
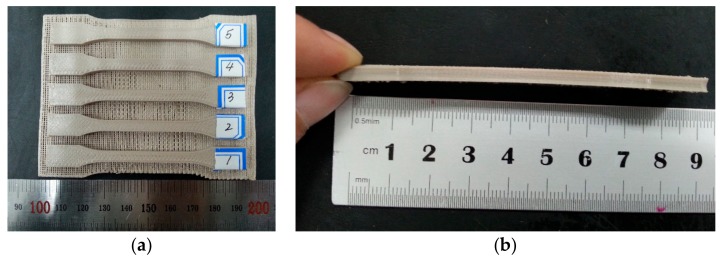
Tensile bars of Group No. 25: (**a**) from XY direction; (**b**) from Z direction.

The results of the tensile tests of every experimental group are listed in Appendix B. The tensile strength ranged from 45 to 67 MPa, the elongation at break ranged from 2% to 3%, and Young’s modulus ranged around 3.0 GPa for all sets. Figure 3 shows the tensile bars from Experiment No. 25. Table 1 illustrates ANOVA with 95% confidence. The F-ratio revealed that both nozzle and platform temperatures were significantly affected tensile strength, while the chamber temperature was insignificant (F-ratio < F (0.05,112)). Other factors were also identified to make the greatest contribution to tensile strength, which indicated that some important factors were ignored during the experiment. The most likely reason was the fluctuation in filament diameter. To confirm this hypothesis, the filament used in this study was compared with a commercial PEEK filament (Apium PEEK 450 Natural, Apium Additive Technologies GmbH^®^, Karlsruhe, German) in terms of diameter. Firstly, a continuous 120-meter-long filament was selected randomly. The filaments were then hauled at a speed of 6 m/min to pass a laser scanning diameter measuring gauge, and the data was recorded at every 3 s.

**Figure 4:**
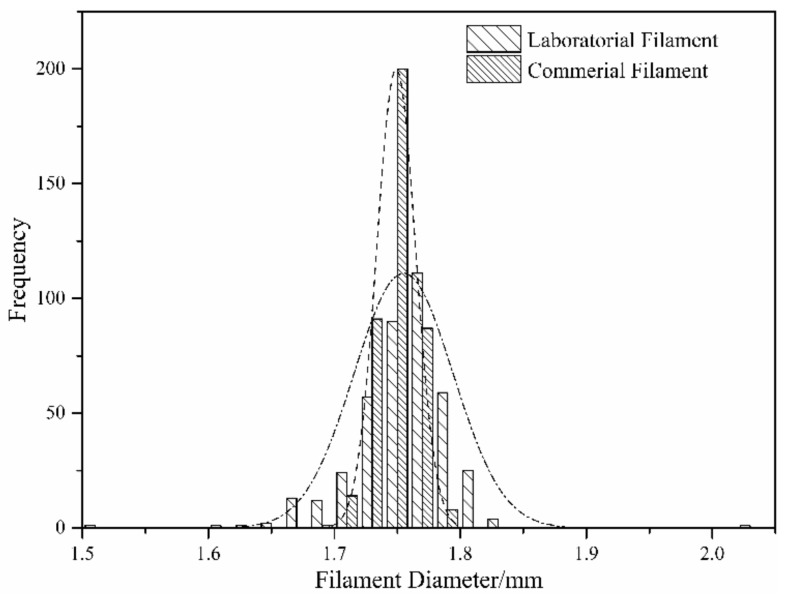
Diameter distribution: the laboratorial filament and commercial filament.

The tests showed that the average diameters of the two filaments were both 1.75 mm. However, a wider range in the laboratorial filament, compared with the commercial filament, was observed, as illustrated in Figure 4. The fluctuating filament resulted in varying the amount of melted material during the printing, which introduced voids in the part and decreased the tensile strength.

**Figure 5:**
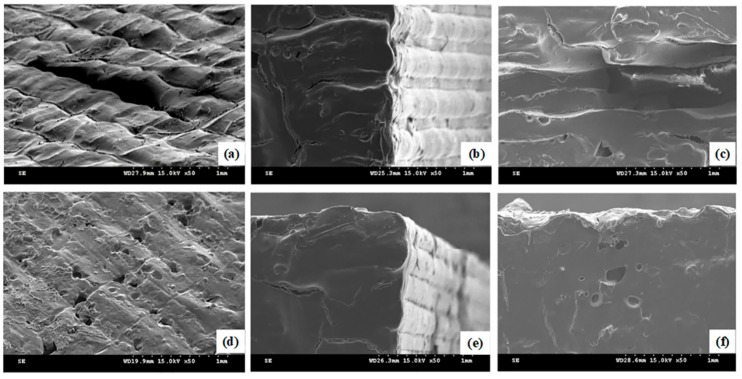
SEM images from different perspectives: (**a**,**d**) layer surface view; (**b**,**e**) isometric view; (**c**,**f**) facture section view; (**a**,**b**,**c**) the samples with the maximum tensile strength; (**d**,**e**,**f**) the samples with the minimum tensile strength.

Samples with the minimum (Group No. 4, printed at $T_{N}$ = 355 °C, $T_{P}$ = 260 °C, and $T_{E}$ = 160 °C) and maximum (Group No. 25, printed at $T_{N}$ = 375 °C, $T_{P}$ = 270 °C, and $T_{E}$ = 160 °C) tensile strengths were examined, both the fracture section and the layer surface, via scanning electron microscope. Clear borders, as shown in Figure 5a, were formed on the layer surface due to the poor deposition between the two printing lines. Meanwhile, nearly no borders can be observed in Figure 5d due to an excellent deposition between the adjacent printing lines. Similarly, a more obvious deposited juncture among layers in the fracture section appeared in the poor samples rather than the better ones in Figure 5c,f. Despite having the best strength, voids and crack could not be avoided both at the fracture section and the layer surfaces (Figure 5e,f), which was the main reason that the tensile strength of specimens printed via FDM was 30% lower than injection-molded specimens [13].

**Figure 6:**
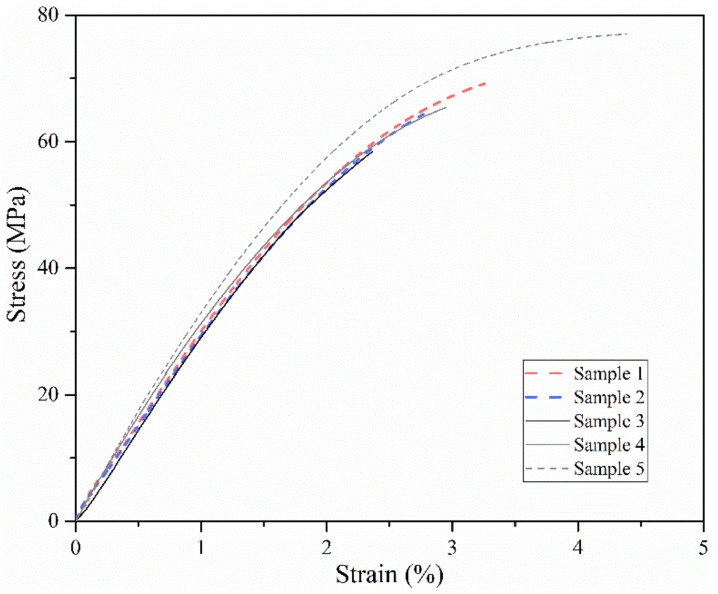
Tensile stress–strain curves of Experiment No. 25.

Figure 6 shows the stress–strain curves from Experiment No. 25. There was a significant drop in elongation when PEEK was fabricated via FDM (2–3%) compared with that fabricated via injection molding (about 45%), which is similar to the results from Valentan and Vaezi [7,10]. The decrease in tensile strength and elongation might be due to the internal defects in the parts. External forces are generally required to avoid voids and defects in traditional fabrication methods, but no such forces are required for FDM. For example, injection pressure (up to 200 MPa), clamp force (typically 50–80 MPa), and holding pressure (10–50 MPa) are encountered in injection molding [14]. Raster angle is the other reason which impacted the strength and elongation. If the raster angle was 0° and/or 180°, the filament was spread along the tensile loading direction, so the loading was borne by the filament itself and the bonding force among the printing lines and layers, which normally results in a slightly higher strength and elongation compared with those at other raster angles. In addition, crystallinity was a sensitive factor for elongation when the raster angle was 0° or 180°. According to Yang et al.’s investigation, when the tensile bars are printed at a 0° raster angle at a suitable nozzle temperature, a low enough crystallinity might result in a more than twofold increase in the elongation compared with injection molding [11].

### 3.2. FTIR

**Figure 7:**
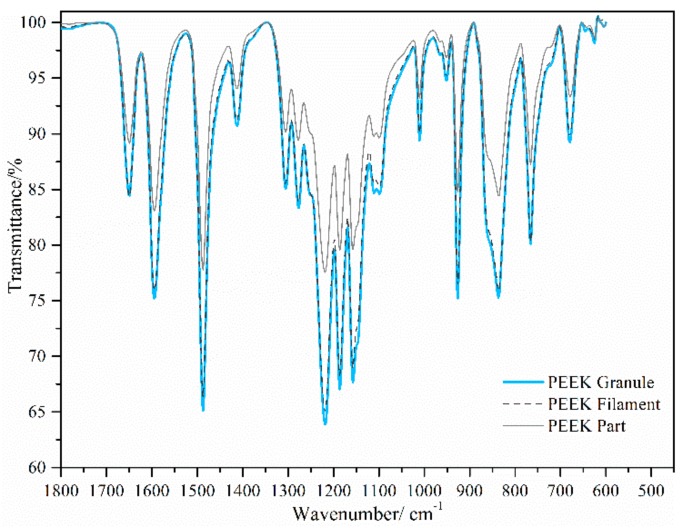
Absorbance spectrum of the PEEK granule, the laboratorial PEEK filament, and the printed PEEK part.

The characteristics of the infrared spectrum of the PEEK granule, filament, and printed samples in the fingerprint region are shown in Figure 7. The bands at 1650 cm^−1^, 1305 cm^−1^, and 926 cm^−1^ indicate the carbonyl stretching vibration, ketone bending motion, and diphenyl ether group features, respectively. The absorption features of ether and diphenyl ether is indicated at bands 1185 cm^−1^ and 1277 cm^−1^. The functional groups in the granule, filament, and printed samples all belonged to PEEK. Thus, neither the thermal heating, the extrusion, nor the high temperature printing changed the structure of PEEK, which is the main requirement of 3D-printed PEEK parts for medical applications [15,16]. In accordance with the ratio of the height between the bands at 1305 cm^−1^ and 1280 cm^−1^, FTIR is also known to be an effective method for crystallinity measurement [17]. In this way, the degrees of crystallinity of the granule, filament, and samples were nearly the same: 16%, 16%, and 17% with a ±1% measurement error, respectively. As the mechanical strength increased with the growth of crystallinity [18], there was a possible further improvement on the printed samples.

### 3.3. Test for In Vitro Cytotoxicity

**Figure 8:**
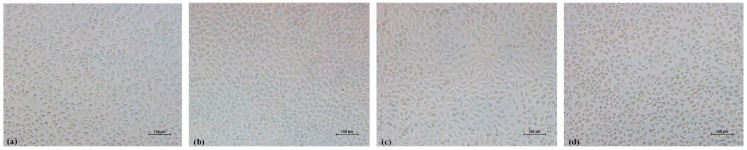
Morphology of cells in (**a**) the blank group, (**b**) the negative group, (**c**) 100% extract, and (**d**) the positive group.

The viability test showed that 98% of cells survived in the culture media with different extract concentrations (98.03 ± 1.79%, 98.70 ± 1.11%, 98.33 ± 1.12%, and 98.43 ± 2.71%, respectively, for 100%, 75%, 50%, and 25% extract), which were similar to the blank (99.17 ± 0.80%) and negative controlled groups (97.3 ± 1.21%). The results were further confirmed by morphology observation as seen in Figure 8. Cells in the blank group, the negative group, and the 100% extract exhibited fibroblastic features, distinct profiles, and significant proliferation. However, plenty of dead cells appeared in the positive group (cell survival rate: 0.6 ± 0.04%). As fibroblasts cells are common in the human body, which are the earliest cells to attach, spread, and proliferate on the implant’s surface [19], PEEK printed via FDM was shown to be promising for medical applications in this respect.

One important limitation should be pointed out here: the materials used in this study were not of medical grade, but of industrial grade. However, no toxic substances were neither produced nor introduced during printing. For a real medical application, a medical-grade PEEK filament must be employed for safety consideration.

## 4. Conclusions

The objective of this study was to preliminarily explore PEEK processed via FDM for medical applications. Mechanical strength, FTIR, and in vitro cytotoxicity tests were chosen as criteria.

Orthogonal experiments and ANOVA analysis revealed that nozzle and printing bed temperatures had significant effects on the tensile strength, while the ambient temperature was relatively insignificant. Thus, a low ambient temperature design can be adopted in a PEEK FDM printer design, as it appeared to facilitate nozzle or platform movement while decreasing the difficulty of the chamber insulation and heat preservation. The experiment also confirmed that the diameter deviation of the filament was a key factor, which should not be ignored during printing. Both the thermal parameters and the filament deviation impacted the interface of the printing lines and layers. An optimized thermo-parameter resulted in improved interlayer bonding and less void in the part, which led to a higher mechanical strength.

The FTIR confirmed that the functional group structures of the filament and the ceiling-printed part were the same as those of the original PEEK granules. In vitro cytotoxicity tests further indicated that no toxic substances were introduced during the printing process.

Although limited tests were carried out in this research, more biological tests should be performed to verify the safety of PEEK fabricated via FDM before 3D-printed PEEK medical devices are used. However, the prospect of such devices is indeed promising.

## Figures and Tables

**Table 1:** Analysis of variance of tensile strength.

Factors	Sum of Squares (SS)	Degrees of Freedom	Mean Square (MS)	F-Ratio	Percent Contribution (P) (100%)
$T_{N}$	490.94	4	122.74	4.07	10.74
$T_{P}$	604.27	4	151.07	5.01	13.22
$T_{E}$	96.78	4	24.20	0.80	2.12
Other/errors	3377.50	112	30.16		73.91
Total	4569.49	124			100

Tabulated F-ratio at 95% confidence level: F (0.05, 120) = 2.45.

## Appendix A

**Table A1:** Group Setting of Orthogonal Experiments.

Experiment No.	Impactor Factors		
	$T_{N}$	$T_{P}$	$T_{E}$
1	355	230	130
2	355	240	140
3	355	250	150
4	355	260	160
5	355	270	170
6	360	230	140
7	360	240	150
8	360	250	160
9	360	260	170
10	360	270	130
11	365	230	150
12	365	240	160
13	365	250	170
14	365	260	130
15	365	270	140
16	370	230	160
17	370	240	170
18	370	250	130
19	370	260	140
20	370	270	150
21	375	230	170
22	375	240	130
23	375	250	140
24	375	260	150
25	375	270	160

## Appendix B

**Table A2:** Results of Orthogonal Experiments.

Experiment No.	Item	Reading 1	Reading 2	Reading 3	Reading 4	Reading 5	Mean
1	Tensile strength/MPa	61.1	57.9	54	62.2	62.6	59.6
	Elongation/%	3	4	2	3	3	3
	Young’s modulus/GPa	2.9	2.7	2.7	3.2	3.1	2.9
2	Tensile strength/MPa	53.7	58.7	54.7	52	48.9	53.6
	Elongation/%	3	3	3	4	4	3
	Young’s modulus/GPa	2.9	3.0	2.9	2.6	2.6	2.8
3	Tensile strength/MPa	64.3	67	63.3	62.3	60.2	63.4
	Elongation/%	3	3	4	3	3	3
	Young’s modulus/GPa	3.2	3.2	3.2	3.1	3.4	3.2
4	Tensile strength/MPa	48.7	41.8	49.5	47.3	44.5	46.4
	Elongation/%	3	2	3	3	3	3
	Young’s modulus/GPa	3.0	2.9	3.0	3.0	2.9	3.0
5	Tensile strength/MPa	46.4	54.9	48.9	52	52.6	51.0
	Elongation/%	2	2	2	2	2	2
	Young’s modulus/GPa	3.1	3.0	3.2	3.1	3.1	3.1
6	Tensile strength/MPa	55.4	54	53	62.9	55.2	56.1
	Elongation/%	3	6	2	6	2	4
	Young’s modulus/GPa	2.9	2.8	3.1	2.7	3.0	2.9
7	Tensile strength/MPa	55.5	57.2	59.8	48.6	52.6	54.7
	Elongation/%	2	3	3	2	2	2
	Young’s modulus/GPa	2.8	3.0	3.1	2.9	3.1	3.0
8	Tensile strength/MPa	46.7	49	50.8	47.9	63	51.5
	Elongation/%	2	2	2	2	2	2
	Young’s modulus/GPa	2.9	3.0	3.2	3.0	3.2	3.1
9	Tensile strength/MPa	48.2	47.4	48.8	45.4	51.4	48.2
	Elongation/%	2	2	2	2	2	2
	Young’s modulus/GPa	3.1	2.9	3.0	2.9	3.0	3.0
10	Tensile strength/MPa	52.8	55.4	53.1	48.9	57.6	53.6
	Elongation/%	2	2	2	2	2	2
	Young’s modulus/GPa	3.1	3.2	3.1	3.1	3.2	3.1
11	Tensile strength/MPa	50.7	53	58.3	53.2	49.7	53.0
	Elongation/%	3	3	3	2	2	3
	Young’s modulus/GPa	2.8	2.7	2.9	2.7	3.0	2.8
12	Tensile strength/MPa	55.7	48.7	57.4	48.7	52.1	52.5
	Elongation/%	2	2	2	2	2	2
	Young’s modulus/GPa	2.7	2.8	2.7	2.8	3.0	2.8
13	Tensile strength/MPa	58.2	56.6	54.9	52.5	52.8	55.0
	Elongation/%	2	2	2	2	2	2
	Young’s modulus/GPa	3.1	3.1	3.1	2.9	3.2	3.1
14	Tensile strength/MPa	60	58.2	63.1	46.9	53.9	56.4
	Elongation/%	2	2	2	2	2	2
	Young’s modulus/GPa	3.3	3.4	3.6	3.3	3.4	3.4
15	Tensile strength/MPa	48.4	66.1	55.8	50.8	53.7	55.0
	Elongation/%	2	2	2	2	2	2.0
	Young’s modulus/GPa	2.9	2.9	3.1	2.9	3.1	3.0
16	Tensile strength/MPa	58.3	54.1	55.8	48.4	51.5	53.6
	Elongation/%	3	3	2	2	2	2
	Young’s modulus/GPa	3.0	3.0	3.0	2.9	3.0	3.0
17	Tensile strength/MPa	49.2	49	59.3	50.4	51.9	52.0
	Elongation/%	2	2	2	2	2	2
	Young’s modulus/GPa	2.90	2.90	3.00	2.90	3.00	2.9
18	Tensile strength/MPa	56.4	50.2	56.3	53.4	49.5	53.2
	Elongation/%	2	2	2	2	2	2
	Young’s modulus/GPa	3.0	3.1	3.2	2.9	3.0	3.0
19	Tensile strength/MPa	49.7	51.3	46.4	42.3	49.6	47.9
	Elongation/%	2	2	2	2	2	2
	Young’s modulus/GPa	2.9	3.1	3.1	2.9	3.1	3.0
20	Tensile strength/GPa	51.7	52.4	44.2	48.3	55.3	50.4
	Elongation/%	2	2	2	2	2	2
	Young’s modulus/GPa	3.0	2.9	3.2	3.1	3.2	3.1
21	Tensile strength/MPa	54	71.5	60	60.3	59.7	61.1
	Elongation%	3	4	3	3	3	3
	Young’s modulus/GPa	2.9	3.1	3.1	2.9	3.0	3.0
22	Tensile strength/MPa	60.1	57.9	51	48.2	52	53.8
	Elongation/%	2	2	2	2	2	2
	Young’s modulus/GPa	3.2	3.2	3.0	2.9	2.9	3.0
23	Tensile strength/MPa	46.4	55.3	49.1	49.4	59.34	51.9
	Elongation/%	2	3	2	2	2	2
	Young’s modulus/GPa	2.7	2.7	2.9	2.9	2.9	2.8
24	Tensile strength/MPa	49.6	54.4	51	52.4	55.4	52.6
	Elongation/%	2	3	2	2	2	2
	Young’s modulus/GPa	3.2	3.0	3.0	3.0	3.2	3.1
25	Tensile strength/MPa	69.2	64.3	58.4	66.3	77	67.0
	Elongation/%	3	3	2	3	4	3
	Young’s modulus/GPa	3.0	2.9	3.0	3.1	3.1	3.0
